# Targeted Nanopore Sequencing for Molecular Characterization of First-Line Drug Resistance and Treatment Outcomes Among Patients in the Republic of Congo

**DOI:** 10.3390/ijms27208999

**Published:** 2026-10-11

**Authors:** Mita Naomie Merveille Dello, Freisnel Hermeland Mouzinga, Breli Bonheur Ngouama, Darrel Ornelle Elion Assiana, Dechvie Jeannis Melissa Boumpouthoud, Jean Claude Djontu, Claujens Chastel Mfoutou Mapanguy, Emmanuel Fonfon Ebata-Mboussa, Beny Erudit Elenga, Jabar Babatunde Pacôme Achimi Abdul, Salomon Bonsi Tchuandom, Franck Hardain Okemba-Okombi, Michel Illoye Ayet, Etienne Nguimbi, Alain Maxime Mouanga, Ayola Akim Adegnika, Francine Ntoumi

**Affiliations:** 1 Fondation Congolaise pour la Recherche Médicale, Brazzaville P.O. Box 2672, Congo; mitanaodello@gmail.com (M.N.M.D.); brelingouama38@gmail.com (B.B.N.); jeannismelissa@gmail.com (D.J.M.B.); cdjontu@yahoo.fr (J.C.D.); chastelmapanguy@gmail.com (C.C.M.M.); 2 Faculté des Sciences et Techniques, Université Marien Ngouabi, Brazzaville P.O. Box 69, Congo; eliondarrel@gmail.com (D.O.E.A.); elengabeny3001@gmail.com (B.E.E.); etienne.ng1612@gmail.com (E.N.); 3 Programme National de Lutte Contre la Tuberculose, Brazzaville P.O. Box 1066, Congo; emmanuelebata08@gmail.com (E.F.E.-M.); salomon.tchuandom.bonsi@undp.org (S.B.T.); franckokemba@gmail.com (F.H.O.-O.); 4 Laboratoire National de Référence des Mycobactéries, Brazzaville P.O. Box 1066, Congo; 5 Faculté des Sciences de la Santé, Université Marien Ngouabi, Brazzaville P.O. Box 2672, Congo; amouanga@gmail.com; 6 Centre de Recherches Médicales de Lambaréné, Lambaréné P.O. Box 1437, Gabon; pacomeabdul@gmail.com (J.B.P.A.A.); ayola-akim.adegnika@uni-tuebingen.de or aadegnika@cermel.org (A.A.A.); 7 Service de Pneumologie, Centre Hospitalier Universitaire, Brazzaville P.O. Box 32, Congo; 8 Centre Antituberculeux de Brazzaville, Brazzaville P.O. Box 1066, Congo; illoye@aol.fr; 9 Société Congolaise de Santé Mentale (SOCOSAM), Brazzaville P.O. Box 2672, Congo; 10 Institute of Tropical Medicine, University of Tübingen, Wilhelmstraße 27, 72074 Tübingen, Germany; 11 German Center for Infection Research, Tübingen Site, University of Tübingen, Wilhelmstraße 27, 72074 Tübingen, Germany

**Keywords:** tuberculosis, drug resistance, *Mycobacterium tuberculosis* complex, treatment outcomes, Republic of Congo, nanopore sequencing

## Abstract

Tuberculosis (TB) remains a major global public health concern, further complicated by the emergence and transmission of drug-resistant strains. Diversity within the Mycobacterium tuberculosis complex (MTBC) may also influence disease progression and treatment response. We conducted a longitudinal study among 126 Xpert MTB/RIF Ultra-confirmed TB patients enrolled and followed at the Antituberculosis Center of Brazzaville between July 2023 and November 2024. MTBC species were identified using the Genotype MTBC assay, and resistance-associated mutations in *rpoB*, *katG*, and *embB* were characterized by targeted Oxford Nanopore sequencing and interpreted using the WHO mutation catalogue and TB-Profiler. The overall treatment success rate was 81.7% (103/126). Among 109/126 samples successfully typed, *M. tuberculosis* accounted for 92.7% (101/109), *Mycobacterium africanum* for 3.7% (4/109), while mixed infection accounted for 0.9% (1/109) and inconclusive profiles for 2.8% (3/109). Resistance-associated mutations were assessed in 65 of the 126 enrolled patients with high-quality sequencing data. Among these 65 isolates, 32.3% (21/65) harbored resistance to at least one of the targeted first-line drugs, including isoniazid (23.1%; 15/65), rifampicin (15.4%; 10/65), and ethambutol (9.2%; 6/65). Multidrug-resistant TB was identified in 12.3% (8/65). The predominant mutations occurred at *rpoB* codon 435, *katG* codon 315, and *embB* codon 306. Treatment success was lower among patients infected with strains carrying a resistance-associated mutation (71.4%). Isoniazid resistance was associated with reduced odds of treatment success in unadjusted analysis (OR = 0.22, 95% CI: 0.05–0.93; *p* = 0.04). These findings suggest a considerable burden of genotypically predicted first-line drug resistance and support expanding molecular drug-susceptibility testing beyond rifampicin in TB surveillance and patient management in the Republic of Congo.

## 1. Introduction

Tuberculosis (TB) is an infectious disease caused by members of the *Mycobacterium tuberculosis* complex (MTBC), which remains the leading cause of death from an infectious disease worldwide [1]. An estimated 10.7 million new TB cases and 1.23 million deaths were reported in 2024 [1].

The MTBC includes several species that are pathogenic to humans, notably *Mycobacterium tuberculosis*, *Mycobacterium africanum*, and *Mycobacterium bovis*, which differ in virulence, geographical distribution, and clinical characteristics [2,3]. Despite the availability of effective treatment regimens, TB continues to represent a major public health challenge. Standard drug-susceptible TB treatment consists of a six-month multidrug regimen combining isoniazid, rifampicin, pyrazinamide, and ethambutol during the intensive phase, followed by isoniazid and rifampicin during the continuation phase [4].

The emergence and spread of drug-resistant tuberculosis (DR-TB), particularly multidrug-resistant TB (MDR-TB), threaten progress toward global TB control. MDR-TB is defined as resistance to both rifampicin and isoniazid. Drug-resistant TB is associated with longer treatment, higher costs, greater toxicity, and poorer clinical outcomes [1,5,6].

Resistance in *Mycobacterium tuberculosis* is primarily driven by spontaneous chromosomal mutations selected under drug pressure. Mutations in the *rpoB* gene are strongly associated with rifampicin resistance, while mutations in the *katG* gene are a major mechanism of isoniazid resistance and mutations in the *embB* gene are linked to ethambutol resistance [7,8,9]. Molecular characterization of these mutations is therefore essential for early detection of resistance and for timely optimization of patient management.

MTBC species diversity may also influence disease progression and therapeutic response. *Mycobacterium africanum*, which is most frequently reported in West Africa, has been associated with slower disease progression compared with *M. tuberculosis* [10,11]. However, data on MTBC species distribution and its clinical relevance remain limited in many African countries, including the Republic of Congo.

Recent advances in molecular diagnostics, including next-generation sequencing, have improved the detection of resistance-associated mutations and strengthened understanding of TB molecular epidemiology. Oxford Nanopore Technologies (ONT) sequencing offers a rapid and increasingly accessible platform that may be particularly valuable for resistance surveillance in resource-limited settings [12,13].

In the Republic of Congo, data remain scarce regarding MTBC species distribution, resistance-associated mutations, and their potential impact on treatment outcomes. This study therefore aimed to (i) characterize MTBC species distribution, (ii) describe resistance-associated mutations in *rpoB*, *katG*, and *embB* genes by ONT sequencing, and (iii) assess the association between first-line TB drug resistance and treatment outcomes among TB patients in Brazzaville, Republic of Congo.

## 2. Results

### 2.1. General Characteristics of the Study Population (N = 126)

A total of 126 patients were included in the study (Table 1). The cohort was predominantly composed of adults aged 20–45 (72.2%), male (66.7%) and single (74.7%). Most participants had primary or secondary education (76.1%) and worked in the informal sector (57.1%). Alcohol consumption was reported in 60.3% of patients. Clinically, most patients were new TB cases (90.5%), and 5.6% were HIV-positive.

### 2.2. Treatment Outcomes of Patients with Tuberculosis

**Figure 1:**
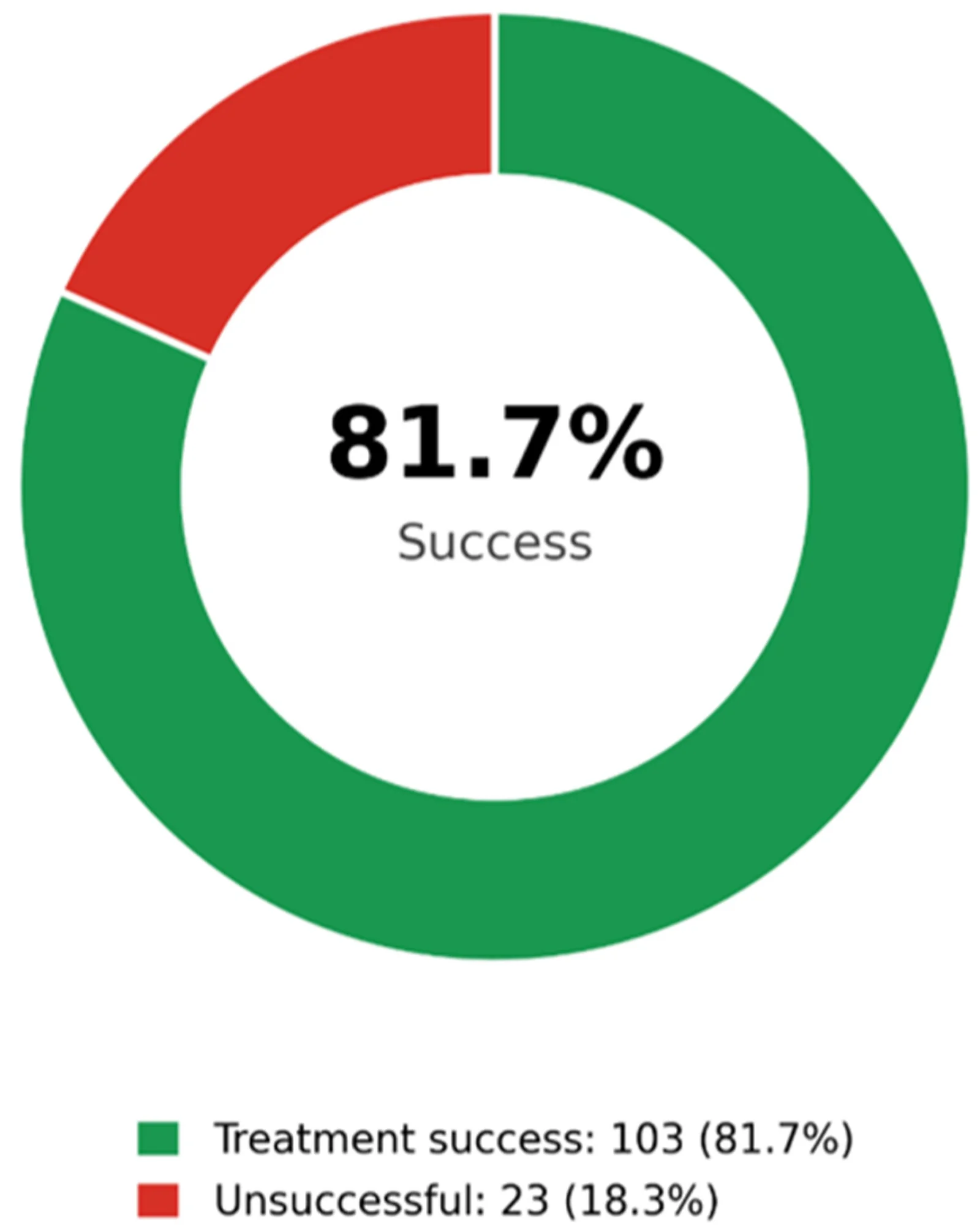
Proportion of successful versus unsuccessful TB treatment outcomes among the 126 enrolled patients. Treatment success comprises cured and treatment-completed cases; unsuccessful outcomes comprise loss to follow-up, death, and treatment failure (n = 126).

**Figure 2:**
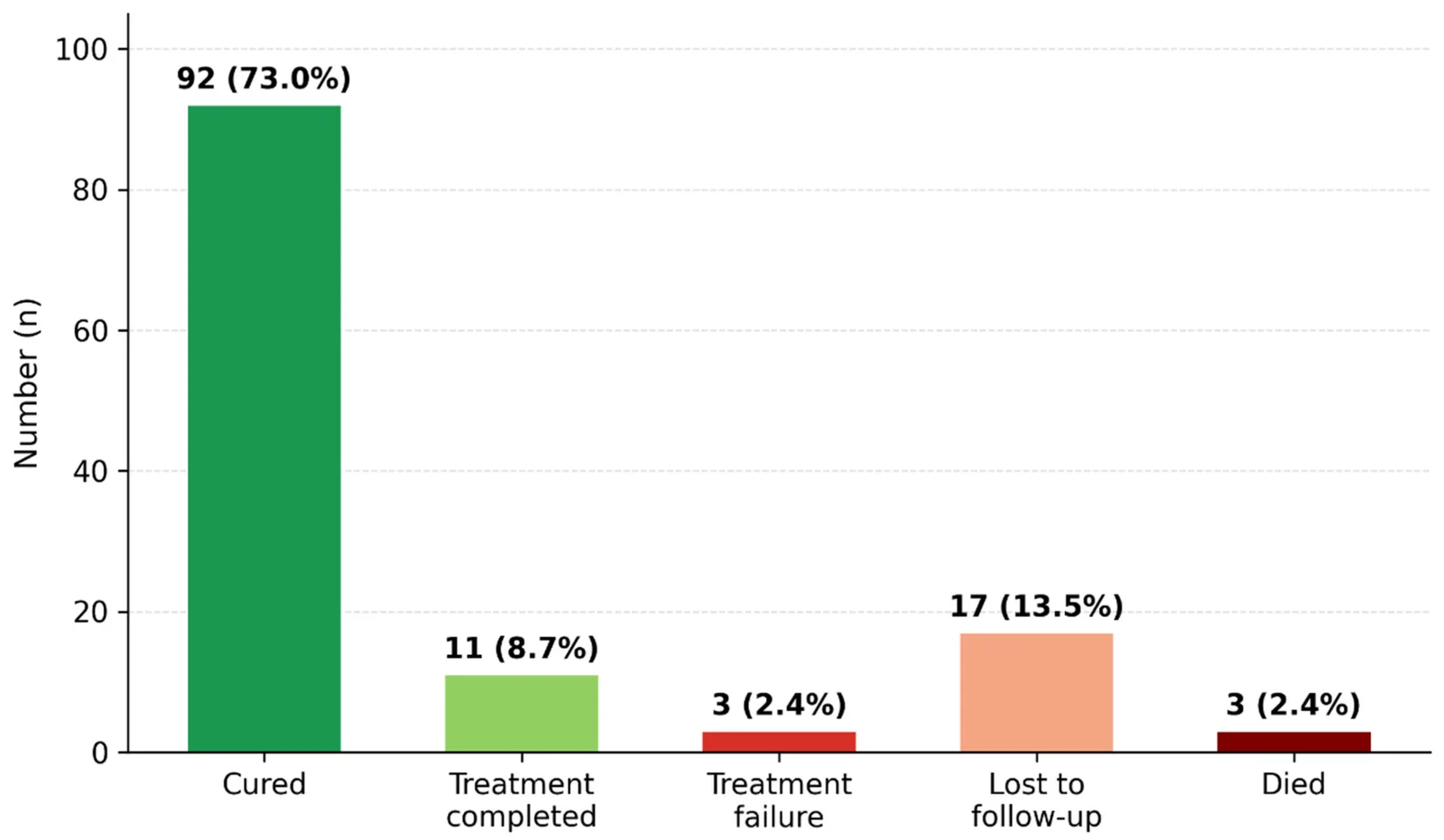
Detailed breakdown of treatment outcomes among the 126 enrolled patients, classified as cured, treatment completed, lost to follow-up, death, or treatment failure, according to WHO operational definitions.

The overall treatment success rate was 81.7% (103 of 126), including 73.0% (92/126) cured patients and 8.7% (11/126) who successfully completed treatment (Figure 1 and Figure 2). Overall, 18.3% (23/126) experienced unsuccessful outcomes, including 13.5% (17/126) lost to follow-up, 2.4% (3/126) deaths, and 2.4% treatment failures (3/126).

### 2.3. Factors Associated with Treatment Success Among Tuberculosis Patients

Associations between treatment success and selected demographic and clinical characteristics were assessed (Table 2). None of the variables evaluated were significantly associated with treatment success (all *p* > 0.05).

### 2.4. Species Distribution

**Figure 3:**
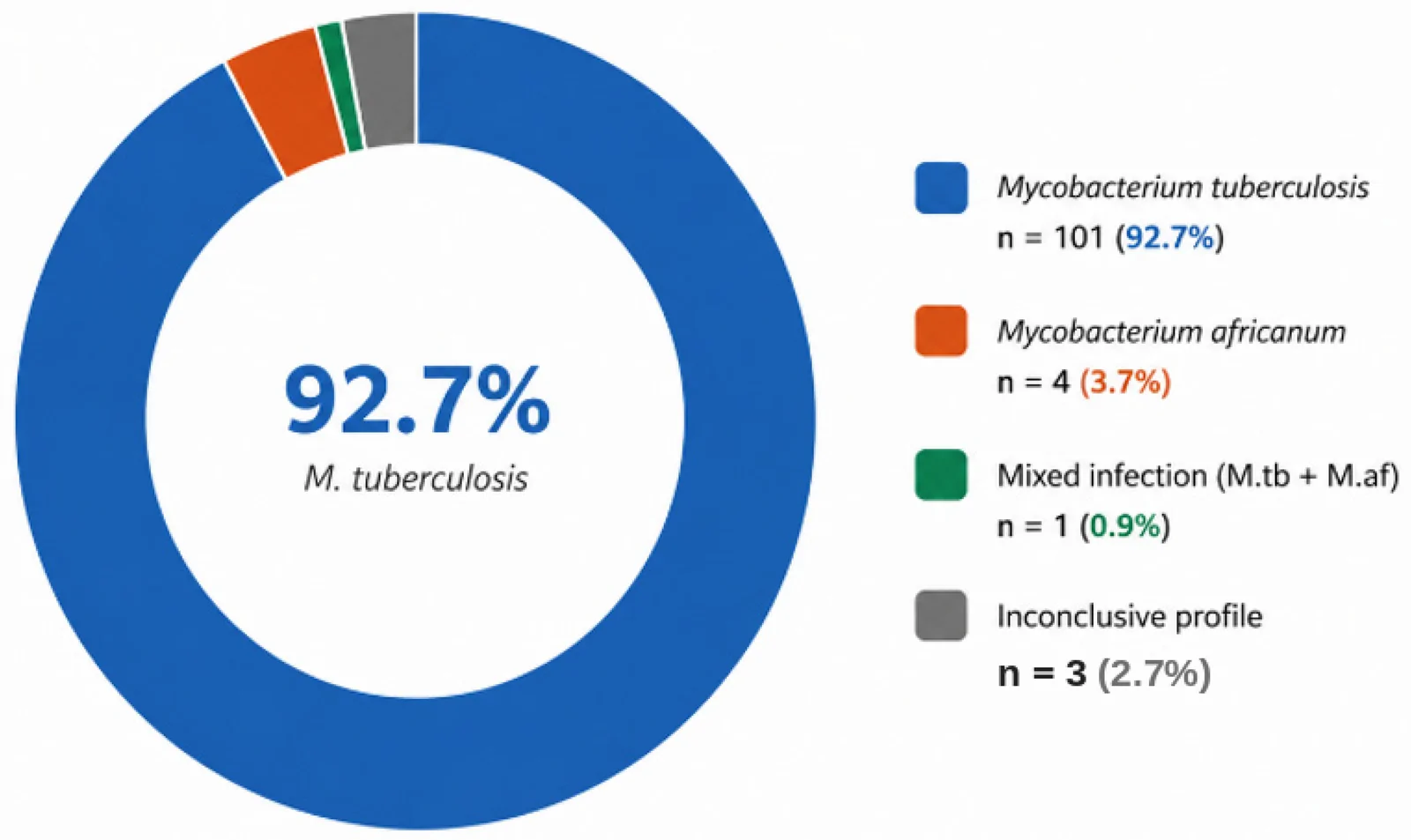
Distribution of Mycobacterium tuberculosis complex species among the 109 successfully genotyped isolates, identified by the GenoType MTBC assay.

Among the 126 participants included, 109 were successfully genotyped for MTBC species identification. *Mycobacterium tuberculosis* predominantly accounted for 92.7% (101/109) of cases. *Mycobacterium africanum* was identified in 3.7% (4/109) of cases. One patient (0.9%) had a mixed infection involving both *M. tuberculosis* and *M. africanum*. while 2.8% (3/109) of isolates yielded indeterminate species profiles (hybridization was technically valid (conjugate and amplification controls positive), but the resulting band pattern could not be assigned to a single recognized MTBC species with sufficient confidence (Figure 3).

### 2.5. Treatment Outcomes by Species

Since *M. tuberculosis* accounted for most genotyped isolates (92.7%, 101/109), treatment outcomes in this group closely mirrored those described for the overall study population. Among the remaining species, all four participants infected with *M. africanum* achieved treatment success (4/4, 100%), as did the participant with mixed *M. tuberculosis*/*M. africanum* infection (1/1). Among the three participants with inconclusive species identification, two were cured, and one died.

### 2.6. Resistance to First-Line Anti-Tuberculosis Drugs

Of the 126 samples included in the study, 79 were culture-positive and entered the ONT sequencing workflow. Following PCR amplification, 73 samples were successfully sequenced, and eight were subsequently excluded after quality filtering, leaving 65 isolates for the final resistance analysis. Of the 65 MTBC isolates included in the analysis, 21 (32.3%) carried mutations associated with resistance to at least one first-line drug.

Based on genotypically predicted resistance, mutations associated with isoniazid resistance were detected in 15 isolates (23.1%), resistance to rifampicin in 10 isolates (15.4%), and resistance to ethambutol in 6 isolates (9.2%). Eight isolates (8/65; 12.3%) showed resistance to both isoniazid and rifampicin and were therefore classified as multidrug-resistant (MDR-TB). Detailed resistance profiles are presented in Table 3.

Table 4 presents the distribution of mutations identified in the *rpoB*, *katG*, and *embB* genes associated with resistance to rifampicin, isoniazid, and ethambutol, respectively. For rifampicin, mutations were mainly observed at codons 435 and 430 of the *rpoB* gene, with p.Asp435Tyr being most frequent, followed by p.Asp435Val and p.Leu430Pro. Isoniazid resistance was exclusively associated with the p.Ser315Thr mutation in the *katG* gene. For ethambutol, most mutations occurred at codon 306 of the *embB* gene, with a predominance of the p.Met-Val substitution.

Following WHO catalogue classification, five of the ten rifampicin-resistant isolates carried p.Asp435Tyr and two carried p.Leu430Pro, both graded as borderline or low-level resistance mutations, while three isolates carried p.Asp435Val, a high-level resistance mutation.

Concordance between baseline Xpert MTB/RIF Ultra and *rpoB* sequencing was assessed among the 65 sequenced isolates. All isolates identified as rifampicin-resistant by Xpert (n = 8) were confirmed resistant by sequencing. Two additional isolates, both carrying the *rpoB* Asp435Tyr mutation, were resistant by sequencing but had not been detected as resistant by baseline Xpert testing.

Of the five non-*M. tuberculosis* isolates identified in this cohort (four *M. africanum*, one mixed infection), two were among the 65 isolates with sequencing data; both had no resistance-associated mutation detected in the three drugs tested. To assess the representativeness of this subset, we compared the 65 sequenced isolates with the 61 non-sequenced patients across baseline demographic and clinical characteristics as well as treatment outcome and baseline xpert MTB/RIFN RR status. No significant differences were observed for sex, age, HIV status, alcohol consumption, baseline Xpert MTB/RIF Ultra rifampicin-resistant status or treatment success. Retreatment cases, however, were less frequently represented among sequenced isolates, with only 25.0% (3/12) of retreatment patients successfully sequenced compared with 54.4% (62/114) of new cases (*p* = 0.070; Supplementary Table S1).

### 2.7. Factors Associated with Resistance (N = 65)

Factors associated with predicted resistance to first-line anti-tuberculosis drugs were analyzed among sequenced isolates. None of the evaluated factors (age, sex, HIV status, TB history, or alcohol consumption) was significantly associated with drug resistance. Retreatment cases had a non-significant trend toward higher resistance than new cases (66.7% vs. 30.6%, OR 4.5, 95% CI 0.4–66.8, *p* = 0.24) (Table 5).

### 2.8. Drug Resistance and Treatment Success

The relationship between first-line drug resistance profiles and treatment success was evaluated among isolates with complete resistance testing. Treatment success was lower among patients infected with strains resistant to at least one drug than among those with no resistance-associated mutation detected in the targeted region (71.4% vs. 90.9%). Isoniazid resistance was inversely associated with treatment success (OR 0.22, 95% CI 0.05–0.93, *p* = 0.04) and was the only factor reaching statistical significance, while treatment success was numerically lower among patients with any resistance-associated mutation, without reaching statistical significance (*p* = 0.06). The distribution of unsuccessful outcomes by INH resistance status was as follows: resistant, 5/15 (33.3%); susceptible, 5/50 (10.0%). Rifampicin, ethambutol, and MDR-TB status did not show significant associations. Of the 15 isoniazid-resistant isolates, 8 were also rifampicin-resistant and received an MDR-TB regimen; the remaining 7 were isoniazid-mono-resistant and remained on the standard first-line regimen, as baseline Xpert MTB/RIF Ultra does not detect isoniazid resistance (Table 6).

## 3. Discussion

This study provides integrated molecular and clinical evidence on TB in the Republic of Congo by combining MTBC species identification, targeted nanopore sequencing of first-line resistance-associated genes, and treatment outcome assessment. The cohort was predominantly composed of young male adults and new TB cases, with a relatively low proportion of HIV co-infection. While these characteristics are consistent with the epidemiology of TB in many low-resource settings, the potentially high burden of resistance patterns and its association with treatment outcomes highlight important programmatic challenges [1,6].

The overall treatment success rate of 81.7% is encouraging and broadly consistent with studies conducted in sub-Saharan Africa, including in the Republic of Congo, where reported success rates generally range from 75% to 85%. However, this rate falls below the expected World Health Organization threshold of 90% [13,14]. The distribution of unsuccessful outcomes suggests that the major challenge is patient retention rather than therapeutic inefficacy. Loss to follow-up accounted for 13.5% of all patients and represented nearly three-quarters of all unsuccessful outcomes, whereas treatment failure and death each accounted for only 2.4%. This suggests that therapeutic regimens may be effective when treatment is completed, although outcomes among patients lost to follow-up remain unknown. Strengthening patient retention and expanding molecular resistance testing are likely complementary priorities for improving TB outcomes in this setting. Species identification revealed *Mycobacterium tuberculosis* as the predominant pathogen (92.7%), while *Mycobacterium africanum* was identified in 3.7% of cases. These findings are consistent with previous literature, as *M. africanum* is traditionally more frequently reported in West Africa and less frequently reported in Central Africa, where *M. tuberculosis* remains the dominant species [12,15,16,17]. Nevertheless, the presence of *M. africanum*, although limited, is consistent with regional data indicating that this species continues to circulate at low frequency in Central Africa [18]. *M. africanum* has been associated with slower growth rates and potentially distinct clinical presentations, and it may remain underdiagnosed in settings relying primarily on phenotypic diagnostic methods [19].

The resistance analysis identified a considerable frequency of resistance-associated mutations to first-line anti-tuberculosis drugs among sequenced isolates. Nearly one-third of sequenced isolates (32.3%) exhibited resistance to at least one drug, a clinically significant finding that suggests active circulation of resistant strains within the population. Resistance was highest for isoniazid (23.1%), followed by rifampicin (15.4%) and ethambutol (9.2%). These observations are consistent with previous reports identifying isoniazid as one of the drugs most frequently affected by resistance in tuberculosis [9,12]. Multidrug-resistant tuberculosis (MDR-TB) was identified in 12.3% of isolates, representing a relatively high prevalence, particularly in a population composed predominantly of new cases. Since MDR-TB is generally more common among retreatment cases, this finding may suggest ongoing community transmission of resistant strains rather than resistance acquired during treatment alone, a hypothesis requiring confirmation by strain-typing studies [6,9,12].

A notable finding of this study is the association between isoniazid resistance and poor treatment outcomes. Among the 65 isolates successfully sequenced, resistance to isoniazid was detected in 15 isolates (23.1%). Patients infected with isolates resistant to isoniazid had lower treatment success rates (66.7%) than patients infected with isoniazid-susceptible isolates. This corresponded to roughly 78% lower estimated odds of treatment success in unadjusted analysis. Among the 65 sequenced isolates, the 10 unsuccessful outcomes comprised 8 losses to follow-up, 1 death, and 1 documented treatment failure; 5 of these patients were isoniazid-resistant, including 1 with combined isoniazid, rifampicin, and ethambutol resistance. Per WHO guidelines, confirmed rifampicin-susceptible, isoniazid-resistant TB (Hr-TB) warrants a modified regimen (rifampicin, ethambutol, pyrazinamide, and levofloxacin for 6 months) rather than the standard first-line regimen. This modified regimen, however, requires molecular confirmation of isoniazid resistance at the point of care, which was unavailable in this setting; baseline Xpert MTB/RIF Ultra does not detect isoniazid resistance. Consequently, 7 of the 15 isoniazid-resistant patients in this cohort had their resistance go undetected at treatment initiation and remained on the standard first-line regimen by default, reflecting a diagnostic gap rather than a guideline-concordant management decision. As loss to follow-up predominates within this composite outcome, we cannot exclude non-biological factors such as access to care, cost, or patient-perceived improvement, which contributed to the observed association, alongside any genuine biological effect of resistance on treatment response. This is particularly concerning given that all isoniazid-resistant isolates carried the *katG* p.Ser315Thr mutation, which is known to confer high-level resistance. This diagnostic gap means that nearly one-quarter of patients in this subgroup may have received suboptimal therapy, with a plausible contribution to unsuccessful treatment outcomes [20]. The association between isoniazid resistance and treatment success, although statistically significant, should be interpreted with appropriate caution given the small number of resistant isolates (n = 15) and the resulting wide confidence interval (0.05–0.93). Confirmation in larger cohorts, ideally with adjustment for retreatment status and other clinical covariates, would strengthen confidence in the magnitude of this effect.

The molecular characterization of resistance-associated mutations demonstrated patterns largely consistent with established global resistance mechanisms [13]. Rifampicin resistance was associated with mutations at codon 435 of the *rpoB* gene, particularly the p.Asp435Tyr, p.Asp435Val and p.Leu430Pro. These mutations occur within the rifampicin resistance-determining region (RRDR) and are known to impair rifampicin binding to RNA polymerase [21,22]. Notably, five of the ten rifampicin-resistant isolates carried p.Asp435Tyr and two carried p.Leu430Pro, both classified as borderline/low-level resistance mutations frequently missed by phenotypic drug susceptibility testing, whereas the three isolates carrying p.Asp435Val, associated with high-level resistance, would likely have been detected by conventional methods. This distinction reinforces the added clinical value of targeted sequencing, which, unlike phenotypic testing, can reliably detect low-level resistance mutations that might otherwise result in undertreatment. Notably, Ser450Leu, the most common rifampicin resistance mutation worldwide, was absent in this cohort despite complete codon coverage, in contrast to our earlier study using the same primers. This apparent shift in mutation spectrum between cohorts collected approximately five years apart warrants confirmation in larger studies.

Similarly, the predominance of *embB*306 mutations among ethambutol-resistant isolates is consistent with previously described molecular mechanisms of ethambutol resistance [21,23]. Mechanistically, the *katG* p.Ser315Thr substitution, carried by all isoniazid-resistant isolates in this cohort, reduces the catalase-peroxidase activity required to convert the isoniazid prodrug into its active form, while retaining sufficient residual enzymatic activity to preserve bacterial fitness, a key reason this mutation predominates globally over catalase-null variants. Similarly, *embB* codon 306 substitutions, predominantly p.Met306Val in this cohort, alter the arabinosyltransferase *EmbB*, reducing ethambutol binding affinity at its target site in arabinogalactan biosynthesis. Together with the RRDR mutations described above for *rpoB*, these findings place the observed mutation spectrum within the established structural and functional basis of first-line drug resistance in *M. tuberculosis.* These findings support the validity of the Oxford Nanopore Technologies (ONT) sequencing approach and confirm that resistance in this setting is primarily mediated through canonical resistance-associated mutations [13].

Comparison between baseline Xpert MTB/RIF Ultra results and *rpoB* sequencing revealed two isolates carrying the p.Asp435Tyr mutation that were not detected as rifampicin-resistant by Xpert, meaning these patients may have received a standard regimen despite true rifampicin resistance. This highlights the potential added value of sequencing as a complement to routine Xpert testing; however, as phenotypic drug susceptibility testing was not performed, genotype-phenotype discordance cannot be excluded, and these two cases are insufficient on their own to establish the superiority of nanopore sequencing over Xpert MTB/RIF Ultra. Clinically, undetected rifampicin resistance carries the risk of inadequate treatment, delayed switch to an appropriate regimen, ongoing bacillary load, and onward transmission of a resistant strain. This discordance is consistent with a known limitation of Xpert probe-based detection for borderline *rpoB* mutations such as p.Asp435Tyr, which produce weaker probe signal shifts than high-confidence resistance mutations. With only two discordant isolates identified here, these findings are illustrative rather than sufficient to estimate how frequently such cases occur in this setting; nonetheless, they support considering reflex sequencing or an equivalent confirmatory test, at least for patients at higher pretest probability of resistance, rather than universal implementation, given the cost and infrastructure constraints of routine nanopore sequencing in this setting.

Although overall drug resistance showed only borderline statistical significance in relation to treatment outcomes (*p* = 0.06), the observed reduction in treatment success remains clinically meaningful. While this precludes any firm conclusion, the direction and magnitude of the effect are consistent with the well-established clinical impact of drug resistance on treatment response, and we consider this finding hypothesis-generating rather than confirmatory. Given the limited number of resistant isolates in our cohort. We propose this association as a candidate for confirmation in larger, multivariable-adjusted studies, rather than dismissing it as a null result. No statistically significant associations were identified for rifampicin resistance, ethambutol resistance, or MDR-TB; however, interpretation is constrained by the small number of resistant isolates within each subgroup.

Several associations that would be biologically plausible were not statistically significant, most notably those involving drug resistance analysis; this may reflect limited statistical power given the small subgroup sample sizes, though this cannot be confirmed with the available data. The association between retreatment status and drug resistance showed a positive trend (OR = 4.5) but did not reach statistical significance (*p* = 0.24), likely because only three retreatment cases were included in the sequencing subset, out of 12 previously treated patients in the overall cohort, limiting generalizability of resistance estimates to this population. Likewise, HIV status was not significantly associated with treatment outcomes, though this is based on only seven HIV-positive patients [24].

Regarding potential selection bias, we compared the 65 sequenced isolates with the 61 non-sequenced patients across baseline demographic and clinical characteristics and treatment outcome. No significant differences were found for sex, age, HIV status, alcohol consumption, or treatment success, indicating the sequenced subset was broadly representative of the cohort. Retreatment history, however, showed a notable imbalance: only 3 of 12 retreatment patients (25.0%) had successfully sequenced samples. Since retreatment status was the strongest correlate of resistance in our cohort, this imbalance suggests our resistance prevalence may underestimate the true burden, as previously treated patients, more likely resistant yet less likely to yield adequate culture or amplifiable DNA, were disproportionately excluded. With only 12 retreatment patients overall, this comparison remains hypothesis-generating and highlights the need for improved diagnostic yield in this subgroup.

Targeted nanopore sequencing offers a favorable balance between diagnostic depth and feasibility in resource-limited settings compared with other resistance-detection methods. Xpert MTB/RIF Ultra provides a turnaround of under two hours but is limited to rifampicin resistance detection and can miss borderline *rpoB* mutations, as illustrated by the discordant isolates identified in this study. Line probe assays (GenoType MTBDRplus) extend coverage to isoniazid resistance with a turnaround of approximately one to two days but interrogate only a limited set of predefined mutation sites. Conventional phenotypic drug susceptibility testing remains the reference standard but requires several weeks and dedicated biosafety infrastructure. Whole-genome sequencing offers the most comprehensive resistance and lineage characterization but requires higher DNA input, greater bioinformatics capacity, and longer turnaround than targeted approaches. Targeted nanopore sequencing, as implemented here, combines a turnaround of approximately 24–48 h from culture-positive isolates with comparatively low equipment cost and portability, making it a feasible intermediate option for strengthening resistance surveillance in similar settings, although its dependence on adequate culture growth or amplifiable DNA remains an important operational constraint, as discussed above.

Several limitations should be acknowledged as noted above. Retreatment cases were underrepresented among sequenced isolates, which may lead to a conservative estimate of the true resistance burden. Second, all associations reported here are unadjusted univariate estimates. The number of outcome events was limited across every analysis in this study, supporting at most one to two covariates under the standard threshold of ten events per covariate, insufficient to adjust simultaneously for age, HIV status, retreatment history, and alcohol use. Residual confounding therefore cannot be excluded, and the association between isoniazid resistance and treatment success in particular should be interpreted with caution given its wide confidence interval.

Third, phenotypic drug susceptibility testing was not performed to confirm the resistance profiles inferred from targeted sequencing. Although mutations were interpreted using the WHO Catalogue of Mutations, genotype-phenotype discordance remains possible for some resistance-associated mutations, and misclassification of resistance status cannot therefore be excluded. Confirmation would further strengthen confidence in the genotype-phenotype correlation. The study did not also evaluate adherence patterns or socioeconomic status, which may influence TB outcomes. Finally, the assay targets limited resistance-determining regions of *rpoB*, *katG*, and *embB* rather than the full genes, and pyrazinamide resistance was not assessed.

Despite these limitations, this study provides valuable molecular data on TB in the Republic of Congo. The findings point to three areas of concern warranting further investigation, including persistent treatment interruption, substantial first-line genotypically predicted drug resistance, and an association between isoniazid resistance and unsuccessful treatment outcome that warrants confirmation given its predominantly loss-to-follow-up composition. Strengthening patient retention, expanding molecular drug-susceptibility testing beyond rifampicin detection, and integrating resistance surveillance into routine TB control activities should be considered important national priorities. Further studies with larger sample sizes and more comprehensive resistance profiling are warranted to confirm and further characterize these findings.

## 4. Materials and Methods

### 4.1. Study Design and Setting

**Figure 4:**
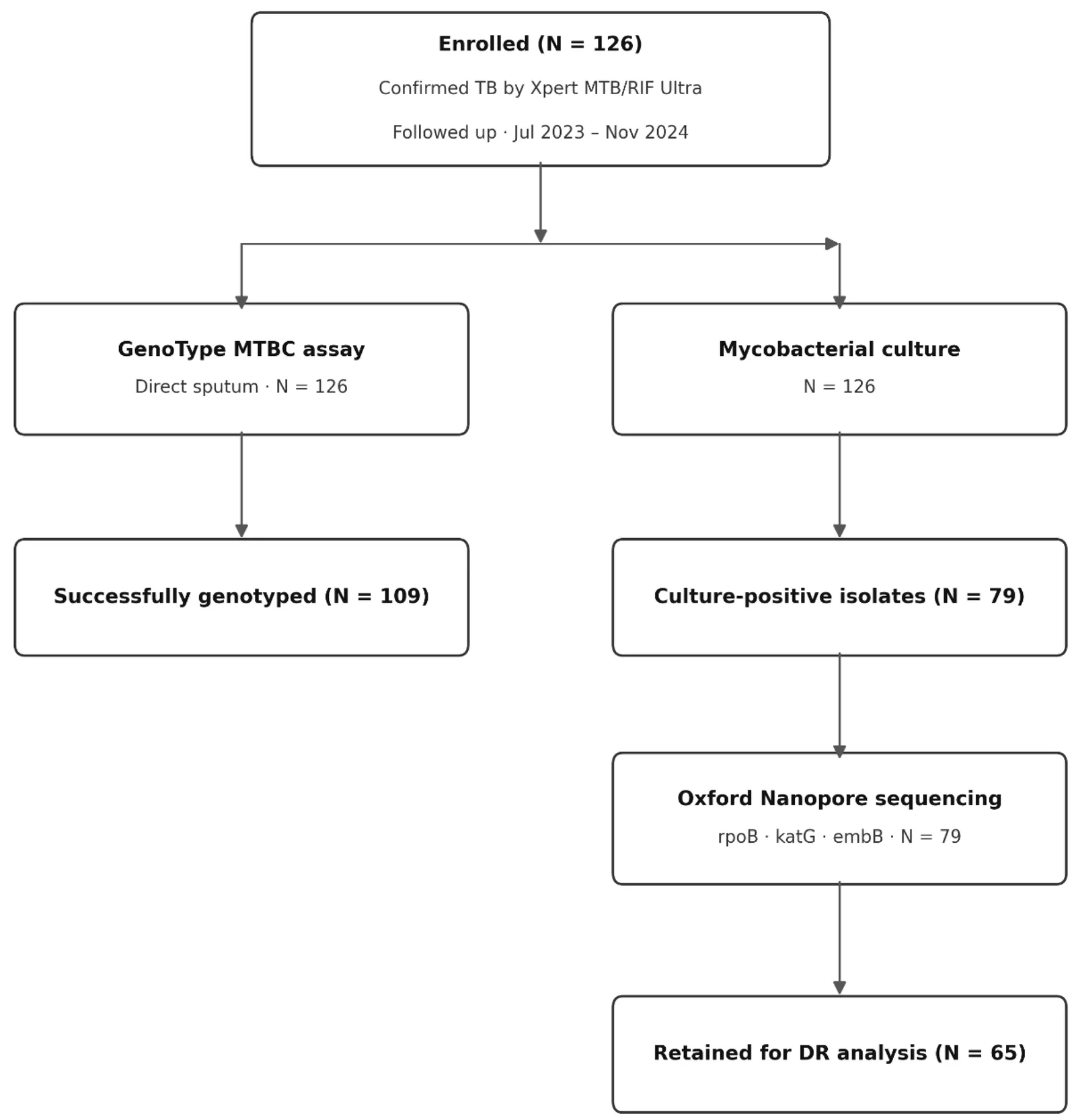
Study population and laboratory workflow. Of the 126 patients included in the study, MTBC species identification was successfully obtained for 109 participants, whereas 17 samples yielded invalid results (no interpretable hybridization profile) despite repeat testing and were excluded from species identification analyses. Among the 79 culture-positive isolates, 65 generated high-quality sequencing data and were included in the analysis of resistance-associated mutations; the remaining 14 isolates were excluded (6 due to PCR amplification failure and 8 due to insufficient read quality after sequencing).

A longitudinal study was conducted among tuberculosis patients at the Antituberculosis Center of Brazzaville in the Republic of Congo from July 2023 to November 2024. Patients were recruited between 13 July and 24 October 2023, with the remaining study period corresponding to treatment follow-up. Patients with confirmed TB based on a positive Xpert MTB/RIF Ultra assay result were eligible for inclusion (Figure 4).

### 4.2. Study Population and Data Collection

A total of 126 patients with confirmed TB were included. Sociodemographic and clinical data, including age, sex, occupation, history of tuberculosis, marital status, alcohol consumption, and smoking habits, were collected using a standardized questionnaire.

### 4.3. Sample Collection, Clinical Follow-Up, Bacteriological Monitoring, and Treatment Regimens

At diagnosis, patients were screened for HIV according to the national HIV testing algorithm using three sequential rapid diagnostic tests: Determine™ HIV-1/2 (Abbott Diagnostics Medical Co., Ltd., Tokyo, Japan) as the screening test, SD Bioline™ HIV-1/2 3.0 (Abbott Diagnostics Korea Inc., Yongin, Republic of Korea) as the confirmatory test, and Uni-Gold™ HIV (Trinity Biotech Manufacturing Ltd., Bray, Ireland) as the tie-breaker test in cases of discordant results. Patients were then initiated on appropriate treatment according to rifampicin resistance status determined by Xpert MTB/RIF Ultra at diagnosis, as per national TB programme guidelines. This routine molecular screening for rifampicin resistance was distinct from the targeted nanopore sequencing performed for research purposes. Drug-susceptible TB cases received the standard WHO-recommended regimen consisting of 2 months of isoniazid, rifampicin, pyrazinamide, and ethambutol, followed by 4 months of isoniazid and rifampicin. Patients with MDR/RR-TB received a 9-month regimen including bedaquiline, levofloxacin, clofazimine, prothionamide, ethambutol, and pyrazinamide.

Sputum samples were collected at predefined treatment time points (months 2, 5, and 6 for drug-susceptible TB patients; monthly for 9 months for RR-TB patients) and examined by Ziehl–Neelsen smear microscopy to inform treatment outcome classification, as defined below.

### 4.4. Operational Definitions

#### Treatment Outcomes

Treatment outcome was defined as the final clinical or bacteriological status recorded after initiation of TB treatment [25].

Treatment completed was defined as completion of treatment without evidence of failure, but without documentation that sputum smear or culture results were negative in the last month of treatment and on at least one previous occasion.

Cured was defined as bacteriologically confirmed TB at treatment initiation with a negative sputum smear or culture result in the last month of treatment and on at least one previous occasion.

Treatment success was defined as the sum of cured and treatment completed cases.

Treatment failure was defined according to WHO criteria applicable to each regimen: for drug-susceptible TB, a positive sputum smear at month 5 or later during treatment; for MDR/RR-TB patients on the 9-month regimen, a positive sputum at month 5 or later, or a regimen change due to lack of bacteriological response or clinical deterioration.

Death was defined as death from any cause before treatment initiation or during the course of TB treatment.

Lost to follow-up was defined as failure to initiate treatment or interruption of treatment for 2 consecutive months or more.

Unsuccessful treatment outcome was defined as the sum of lost to follow-up, death, and treatment failure.

### 4.5. Mycobacterial Culture

Clinical specimens collected from patients with Xpert MTB/RIF Ultra-confirmed TB were processed for mycobacterial culture. Following decontamination with the N-acetyl-L-cysteine-sodium hydroxide (NALC-NaOH) method, specimens were cultured using the BACTEC MGIT 960 system (Becton Dickinson, Franklin Lakes, NJ, USA). Positive cultures were confirmed by Ziehl-Neelsen staining for acid-fast bacilli, screened for contamination by subculture on blood agar, and identified as members of the *Mycobacterium tuberculosis* complex (MTBC) using the Capilia TB-Neo immunochromatographic assay targeting the MPT64 antigen. Only confirmed, uncontaminated MTBC isolates were included in downstream molecular analyses.

### 4.6. DNA Extraction and Amplification

Genomic DNA was extracted from culture-positive isolates using the cetyltrimethylammonium bromide (CTAB) method. Briefly, bacterial pellets were resuspended in 500 µL of TE buffer and treated sequentially with lysozyme (10 mg/mL), 10% sodium dodecyl sulfate, and proteinase K (20 mg/mL). Cell lysis was completed using 2% CTAB, followed by DNA purification with chloroform–isoamyl alcohol (24:1) extraction. DNA was precipitated with isopropanol, washed with 70% ethanol, air-dried, and resuspended in TE buffer before storage at −20 °C until further analysis.

Specific regions of the *rpoB*, *katG*, and *embB* genes were amplified by polymerase chain reaction using gene-specific primers. PCR products were verified by electrophoresis on a 2% agarose gel. DNA concentration, purity and integrity were assessed by spectrophotometry using a BioSpectrometer (Eppendorf AG, Hamburg, Germany).

(Eppendorf, Hamburg, Germany) based on the A260/A280 ratio, and by agarose gel electrophoresis. The primer sequences used are presented in Table 7. Isolates for which PCR amplification was unsuccessful, or which yielded insufficient amplicon concentration for library preparation, were excluded from downstream sequencing.

The rpoB amplicon (158 bp; NC_000962.3 positions 761,059–761,216) spans codons 418–470, fully covering the 81-bp rifampicin resistance-determining region (codons 426–452).

### 4.7. Sequencing and Bioinformatics Analysis

PCR amplicons targeting the *rpoB*, *katG*, and *embB* genes were purified using 1× AMPure XP beads (Beckman Coulter, Brea, CA, USA) and normalized to 100 ng/µL prior to library preparation. Sequencing libraries were prepared using the SQK-RBK114.96 Rapid Barcoding Kit (Oxford Science Park, Oxford, UK)) according to the manufacturer’s instructions, with a minimum of 50 ng of DNA per barcode used before pooling.

Sequencing was performed on an FLO-MIN114 flow cell using R10.4.1 chemistry, on the ONT GridION platform, for 24 h; given the short amplicon targets, the read-length range was set between 50 and 300 bp. Basecalling was performed using Guppy v6.4.2 with the High Accuracy (HAC) model appropriate for R10.4.1 chemistry, and demultiplexing was performed using the barcode information generated during the rapid barcoding workflow.

Raw sequencing data were assessed using NanoPlot (v1.42.0), and reads with a mean quality score < 10 was removed using NanoFilt (v2.8.0). High-quality reads were aligned to the *Mycobacterium tuberculosis* H37Rv reference genome (NC_000962.3) using Minimap2 (v2.28-r1209). The resulting alignment files were sorted and indexed using SAMtools (v1.17). Per-base sequencing depth across each target amplicon was calculated from the sorted BAM files using SAMtools depth. Following quality filtering, the mean sequencing depth across successfully sequenced target amplicons ranged from 5182× to 8227× for *embB*, 1697× to 1852× for *rpoB*, and reached 657× for *katG*. Coverage of ≥30× was achieved across 100% of the target amplicon length in all successfully sequenced amplicons, while ≥100× coverage was achieved across 96.4–99.0% of the target amplicon length, indicating sufficient sequencing depth for reliable downstream variant detection. An amplicon was considered successfully sequenced when quality-filtered reads showed specific alignment to the expected target region. Amplicons without sufficient specific mapping were considered sequencing failures and excluded from downstream variant analysis.

Sequence alignments were visualized using Integrative Genomics Viewer (IGV v2.16.2) to assess coverage and mutation sites. Resistance-associated variants were identified using TB-Profiler v4 with default parameters (minimum depth 10×, minimum variant allele frequency ~10%). The identified variants were interpreted according to the World Health Organization (WHO) 2023 Catalogue of Mutations in *Mycobacterium tuberculosis* Complex and Their Association with Drug Resistance [13].

### 4.8. Species Identification (MTBC)

Species identification within the MTBC was performed on sputum samples collected from the 126 study participants using the GenoType MTBC version 1.x assay (Hain Lifescience GmbH, Nehren, Baden-Württemberg, Germany). Direct application to sputum, rather than exclusively to cultured isolates, has been documented in the literature by Mbelele et al. [26] and reduces culture bias against *M. africanum* as by Sanoussi et al. [27]. The mixed *M. tuberculosis*/*M. africanum* result was independently repeated and confirmed.

Since not all samples were culture-positive and the line probe assay can be performed on either cultured isolates or direct clinical specimens, the analysis was conducted directly on sputum samples regardless of culture status. DNA extraction was carried out using the GenoLyse kit version 1 (Hain Lifescience GmbH, Nehren, Württemberg, Germany) as recommended by the manufacturer. The extracted DNA served as a template for multiplex PCR amplification targeting genomic regions specific to species within the MTBC. The resulting amplicons were then subjected to reverse hybridization on nitrocellulose strips containing immobilized oligonucleotide probes. Hybridization patterns were interpreted using the manufacturer’s reference chart to differentiate MTBC species. Seventeen samples yielded insufficient DNA or uninterpretable hybridization profiles and were therefore excluded from species identification analyses.

### 4.9. Statistical Analysis

The data were analyzed using GraphPad Prism (version 8.0.2). Descriptive statistics were used to summarize the study population and resistance profiles, and categorical variables were presented as frequencies and percentages.

Given the limited number of outcome events across all association analyses (10–23), multivariable adjustment was not feasible under the standard threshold of at least ten events per covariate; all reported associations are therefore unadjusted, assessed using the Chi-square test or Fisher’s exact test, as appropriate.

### 4.10. Ethical Considerations

The study protocol was approved by the Institutional Ethics Committee of the Fondation Congolaise pour la Recherche Médicale (N/Réf:-062-/CEI/FCRM/PR/07BA23). Before enrolment, participants were informed about the purpose of the study and written informed consent was obtained from all participants. For participants younger than 18 years of age, assent was obtained in addition to written consent from parents or legal guardians.

## Figures and Tables

**Table 1:** Socio-demographic, clinical and behavioral characteristics of tuberculosis patients.

Variable	Category	n (%)
Age (years)	<19	13 (10.3)
	20–45	91 (72.2)
	≥46	22 (17.5)
Sex	Male	84 (66.7)
	Female	42 (33.3)
Education level	Primary/Secondary	98 (77.8)
	Higher	28 (22.2)
Occupation	Formal	42 (33.3)
	Informal	72 (57.1)
	Unemployed	12 (9.6)
Marital status	Single	94 (74.7)
	Married/cohabiting	32 (25.3)
Alcohol consumption	Yes	77 (61.1)
	No	49 (38.9)
TB history	New case	114 (90.5)
	Previously treated case	12 (9.5)
HIV status	Positive	7 (5.6)
	Negative	119 (94.4)

**Table 2:** Assessment of risk factors associated with treatment success (N = 126).

Variable	Category	Treatment Success n/N (%)	OR (95% CI)	*p*-Value
Age (years)	<20	10/13 (76.9)	1 (ref)	
	20–45	76/91 (83.5)	1.52 (0.41–5.50)	0.54
	≥46	17/22 (77.3)	1.02 (0.23–5.66)	0.97
Sex	Female	34/42 (81.0)	1 (ref)	
	Male	69/84 (82.1)	1.1 (0.42–2.8)	1.00
Education level	Primary/Secondary	80/98 (81.6)	1 (ref)	-
	Higher	23/28 (82.1)	1.04 (0.36–2.84)	0.99
HIV status	Negative	97/119 (81.5)	1 (ref)	-
	Positive	6/7 (85.7)	1.44 (0.16–12.78)	0.74
TB history	New case	93/114 (81.5)	1 (ref)	-
	Retreatment	10/12 (83.3)	1.12 (0.26–5.46)	0.99
Alcohol consumption	No	37/49 (75.5)	1 (ref)	-
	Yes	66/77 (85.7)	1.94 (0.77–4.86)	0.16

OR (odds ratio); CI (confidence interval); ref (reference category). *p*-values calculated using Fisher’s exact test.

**Table 3:** Drug resistance profile.

Resistance Profile	Effective (n)	Percentage (%)
Overall resistance	21/65	32.3
Isoniazid (INH)	15/65	23.1
Rifampicin (RIF)	10/65	15.4
Ethambutol (EMB)	6/65	9.2
MDR-TB (RIF + INH)	8/65	12.3
RIF + EMB	2/65	3.1
INH + EMB	2/65	3.1
RIF + INH + EMB	2/65	3.1

Single-drug rows include isolates with additional co-resistance to other drugs; the combination rows (RIF + EMB, INH + EMB, RIF + INH + EMB, MDR-TB) indicate the corresponding overlapping subsets. Applying inclusion-exclusion to these counts (15 + 10 + 6 − 8 − 2 − 2 + 2 = 21) reproduces the overall resistance prevalence found.

**Table 4:** Distribution of mutations in *rpoB*, *katG*, and *embB* genes associated with anti-tuberculosis drug resistance.

Drug	Gene	Codon Position	Amino Acid Change	Nucleotide Change	Number of Isolates	Frequency (%)
Rifampicin	*rpoB*	435	p.Asp—Tyr	GAC—TAC	5/10	50.0
		435	p.Asp—Val	GAC—GTC	3/10	30.0
		430	p.Leu—Pro	CTG—CCG	2/10	20.0
Isoniazid	*katG*	315	p.Ser—Thr	AGC—ACC	15/15	100.0
Ethambutol	*embB*	306	p.Met—Ile	ATG—ATA	1/6	16.7
		306	p.Met—Val	ATG—GTG	5/6	83.3

**Table 5:** Assessment of risk factors associated with resistance to first-line anti-tuberculosis drugs among sequenced isolates (N = 65).

Variable	Category	Resistant n/N (%)	OR (95% CI)	*p*-Value
Age (years)	<20	2/6 (33.3)	1 (ref)	—
	20–45	16/48 (33.3)	1.00 (0.16–6.1)	0.99
	≥46	3/11 (27.3)	0.75 (0.09–6.3)	0.99
Sex	Male	13/44 (29.6)	1 (ref)	
	Female	8/21 (38.1)	1.5 (0.5–4.3)	0.57
HIV status	Negative	20/60 (33.3)	1 (ref)	—
	Positive	1/5 (20.0)	0.5 (0.05–5.2)	0.99
TB history	New case	19/62 (30.6)	1 (ref)	—
	Retreatment	2/3 (66.7)	4.5 (0.4–66.8)	0.24
Alcohol consumption	No	9/25 (36.0)	1 (ref)	—
	Yes	12/40 (30.0)	0.76 (0.26–2.20)	0.79

OR, odds ratio; CI, confidence interval; ref, reference category. Analysis was restricted to the 65 isolates with genotypic resistance prediction data. Drug resistance was defined as resistance to at least one first-line anti-tuberculosis drug (isoniazid, rifampicin, or ethambutol).

**Table 6:** Association between first-line TB drug resistance and treatment success among tuberculosis patients with genotypic resistance prediction data (N = 65).

Variable	Category	Treatment Success n/N (%)	OR (95% CI)	*p*-Value
Overall resistance status	No resistance-associated mutation detected	40/44 (90.9)	1 (ref)	
	Any resistance	15/21 (71.4)	0.25 (0.07–0.92)	0.06
Isoniazid resistance	INH susceptible	45/50 (90.0)	1 (ref)	—
	INH resistant	10/15 (66.7)	0.22(0.05–0.93)	0.04
Rifampicin resistance	RIF susceptible	47/55 (85.4)	1 (ref)	—
	RIF resistant	8/10 (80.0)	0.73 (0.13–4.02)	0.65
Ethambutol resistance	EMB susceptible	50/59 (84.7)	1 (ref)	—
	EMB resistant	5/6 (83.3)	0.89 (0.11–7.67)	0.99
Multidrug resistance	Non-MDR-TB	48/57 (84.2)	1 (ref)	—
	MDR-TB (RIF + INH)	7/8 (87.5)	1.31 (0.14–12.0)	1.00

OR, odds ratio; CI, confidence interval; ref, reference category; INH, isoniazid; RIF, rifampicin; EMB, ethambutol; MDR-TB, multidrug-resistant tuberculosis. Analysis was restricted to the 65 isolates with genotypic resistance prediction data. Resistance was defined as resistance to at least one first-line anti-tuberculosis drug.

**Table 7:** Primers used for PCR amplification of resistance-associated genes.

Gene	Primer	Sequence (5′–3′)	Amplicon Size
*rpoB*	Forward	TCGCCGCGATCAAGGAGT	158 bp
	Reverse	GTGCACGTCGCGGACCTCCA	
*katG*	Forward	GAAACAGCGGCGCTGATCGT	210 bp
	Reverse	GTTGTCCCATTTCGTCGGGG	
*embB*	Forward	CCGACCACGCTGAAACTG	250 bp
	Reverse	GTAATACCACCAGCCGAAGGGATCCT	

## Data Availability

Upon request to the corresponding author, raw data will be made available.

## Supplementary Materials

The following supporting information can be downloaded at: https://www.mdpi.com/article/10.3390/ijms27208999/s1.
