# Supplementary material for: Targeted Nanopore Sequencing for Molecular Characterization of First-Line Drug Resistance and Treatment Outcomes Among Patients in the Republic of Congo
Source: Int J Mol Sci. 2026 Oct 11;27(20):8999. doi: 10.3390/ijms27208999 (PMC13704716; doi:10.3390/ijms27208999)
Supplement: Supplementary file 1 [file ijms-27-08999-s001.zip › ijms-4529708-supplementary.pdf]

Supplementary Table S1. Comparison of baseline characteristics and treatment outcome between patients with and without genotypic resistance data.

Variable	Category	Sequenced (n=65)	Non-sequenced (n=61)	OR (95% CI)	p-value
Sex	Male	44/65 (67.7%)	40/61 (65.6%)	1.10 (0.52–2.31)	0.85
Age	≥46 years	11/65 (16.9%)	11/61 (18.0%)	0.93 (0.37–2.32)	1.00
HIV status	Positive	5/65 (7.7%)	2/61 (3.3%)	2.46 (0.46–13.17)	0.44
TB history	Retreatment	3/65 (4.6%)	9/61 (14.8%)	0.28 (0.07–1.09)	0.070
Alcohol consumption	Yes	40/65 (61.5%)	36/61 (59.0%)	1.11 (0.54–2.27)	0.86
Treatment outcome	Success	55/65 (84.6%)	48/61 (78.7%)	1.49 (0.60–3.70)	0.49
Xpert MTB/RIF Ultra RR status	RR	8/65 (12.3%)	5/61 (8.2%)	1.57 (0.64–3.87)	0.56

Supplementary Table S2. Isolate-level mutation, resistance profile, baseline Xpert result, treatment regimen, and outcome data for the 65 sequenced Mycobacterium tuberculosis complex isolates.

ID	*Xpert RIF*	*rpoB mutation*	*rpoB profile*	*katG mutation*	*katG profile*	*embB mutation*	*embB profile*	*Resistance profile*	*Xpert–seq concordance*	*Regimen*	*Outcome*	*Outcome (binary)*
ISO01	Susceptible	-	Sensible	-	Sensible	-	Sensible	None	Concordant (S)	Standard first-line	Died	Unsuccessful
ISO02	Susceptible	-	Sensible	-	Sensible	-	Sensible	None	Concordant (S)	Standard first-line	Cured	Success
ISO03	Susceptible	-	Sensible	-	Sensible	-	Sensible	None	Concordant (S)	Standard first-line	Cured	Success
ISO04	Susceptible	-	Sensible	-	Sensible	-	Sensible	None	Concordant (S)	Standard first-line	Cured	Success
ISO05	Susceptible	-	Sensible	-	Sensible	-	Sensible	None	Concordant (S)	Standard first-line	Lost to follow-up	Unsuccessful
ISO06	Susceptible	-	Sensible	-	Sensible	-	Sensible	None	Concordant (S)	Standard first-line	Cured	Success
ISO07	Susceptible	Asp435Tyr	Résistant	-	Sensible	-	Sensible	RIF-mono	Discordant (missed by Xpert)	Standard first-line	Cured	Success
ISO08	Susceptible	-	Sensible	-	Sensible	-	Sensible	None	Concordant (S)	Standard first-line	Cured	Success
ISO09	Susceptible	-	Sensible	-	Sensible	-	Sensible	None	Concordant (S)	Standard first-line	Cured	Success

ISO10	Susceptible	-	Sensible	-	Sensible	-	Sensible	None	Concordant (S)	Standard first-line	Cured	Success
ISO11	Susceptible	-	Sensible	-	Sensible	-	Sensible	None	Concordant (S)	Standard first-line	Cured	Success
ISO12	Susceptible	-	Sensible	-	Sensible	-	Sensible	None	Concordant (S)	Standard first-line	Cured	Success
ISO13	Susceptible	-	Sensible	-	Sensible	-	Sensible	None	Concordant (S)	Standard first-line	Cured	Success
ISO14	Susceptible	-	Sensible	Ser315Thr	Résistant	-	Sensible	INH-mono	Concordant (S)	Standard first-line	Lost to follow-up	Unsuccessful
ISO15	Susceptible	-	Sensible	-	Sensible	-	Sensible	None	Concordant (S)	Standard first-line	Cured	Success
ISO16	Susceptible	-	Sensible	-	Sensible	-	Sensible	None	Concordant (S)	Standard first-line	Cured	Success
ISO17	Susceptible	-	Sensible	-	Sensible	Met306Ile	Résistant	EMB-mono	Concordant (S)	Standard first-line	Cured	Success
ISO18	Susceptible	-	Sensible	Ser315Thr	Résistant	-	Sensible	INH-mono	Concordant (S)	Standard first-line	Cured	Success
ISO19	Susceptible	Asp435Tyr	Résistant	-	Sensible	-	Sensible	RIF-mono	Discordant (missed by Xpert)	Standard first-line	Treatment failure	Unsuccessful
ISO20	Susceptible	-	Sensible	-	Sensible	-	Sensible	None	Concordant (S)	Standard first-line	Cured	Success
ISO21	Resistant	Asp435Val	Résistant	Ser315Thr	Résistant	Met306Val	Résistant	MDR+EMB (RIF+INH+EMB)	Concordant (R)	MDR-TB regimen	Lost to follow-up	Unsuccessful
ISO22	Susceptible	-	Sensible	-	Sensible	-	Sensible	None	Concordant (S)	Standard first-line	Treatment completed	Success

ISO23	Resistant	Leu450Val	Résistant	Ser315Thr	Résistant	Met306Val	Résistant	MDR+EMB (RIF+INH+EMB)	Concordant (R)	MDR-TB regimen	Treatment completed	Success
ISO24	Susceptible	-	Sensible	-	Sensible	-	Sensible	None	Concordant (S)	Standard first-line	Cured	Success
ISO25	Resistant	Asp435Tyr	Résistant	Ser315Thr	Résistant	-	Sensible	MDR (RIF+INH)	Concordant (R)	MDR-TB regimen	Treatment completed	Success
ISO26	Resistant	Asp435Val	Résistant	Ser315Thr	Résistant	-	Sensible	MDR (RIF+INH)	Concordant (R)	MDR-TB regimen	Cured	Success
ISO27	Susceptible	-	Sensible	-	Sensible	-	Sensible	None	Concordant (S)	Standard first-line	Cured	Success
ISO28	Susceptible	-	Sensible	-	Sensible	-	Sensible	None	Concordant (S)	Standard first-line	Cured	Success
ISO29	Susceptible	-	Sensible	-	Sensible	-	Sensible	None	Concordant (S)	Standard first-line	Lost to follow-up	Unsuccessful
ISO30	Susceptible	-	Sensible	-	Sensible	Met306Val	Résistant	EMB-mono	Concordant (S)	Standard first-line	Treatment completed	Success
ISO31	Susceptible	-	Sensible	-	Sensible	-	Sensible	None	Concordant (S)	Standard first-line	Cured	Success
ISO32	Susceptible	-	Sensible	-	Sensible	-	Sensible	None	Concordant (S)	Standard first-line	Cured	Success
ISO33	Susceptible	-	Sensible	-	Sensible	-	Sensible	None	Concordant (S)	Standard first-line	Cured	Success
ISO34	Resistant	Asp435Tyr	Résistant	Ser315Thr	Résistant	-	Sensible	MDR (RIF+INH)	Concordant (R)	MDR-TB regimen	Cured	Success

ISO35	Susceptible	-	Sensible	Ser315Thr	Résistant	-	Sensible	INH-mono	Concordant (S)	Standard first-line	Lost to follow-up	Unsuccessful
ISO36	Susceptible	-	Sensible	-	Sensible	-	Sensible	None	Concordant (S)	Standard first-line	Cured	Success
ISO37	Susceptible	-	Sensible	-	Sensible	-	Sensible	None	Concordant (S)	Standard first-line	Cured	Success
ISO38	Susceptible	-	Sensible	-	Sensible	-	Sensible	None	Concordant (S)	Standard first-line	Lost to follow-up	Unsuccessful
ISO39	Susceptible	-	Sensible	-	Sensible	-	Sensible	None	Concordant (S)	Standard first-line	Cured	Success
ISO40	Susceptible	-	Sensible	Ser315Thr	Résistant	-	Sensible	INH-mono	Concordant (S)	Standard first-line	Lost to follow-up	Unsuccessful
ISO41	Susceptible	-	Sensible	-	Sensible	-	Sensible	None	Concordant (S)	Standard first-line	Cured	Success
ISO42	Susceptible	-	Sensible	-	Sensible	-	Sensible	None	Concordant (S)	Standard first-line	Cured	Success
ISO43	Susceptible	-	Sensible	-	Sensible	Met306Val	Résistant	EMB-mono	Concordant (S)	Standard first-line	Cured	Success
ISO44	Susceptible	-	Sensible	-	Sensible	-	Sensible	None	Concordant (S)	Standard first-line	Cured	Success
ISO45	Resistant	Leu450Val	Résistant	Ser315Thr	Résistant	-	Sensible	MDR (RIF+INH)	Concordant (R)	MDR-TB regimen	Cured	Success
ISO46	Susceptible	-	Sensible	Ser315Thr	Résistant	-	Sensible	INH-mono	Concordant (S)	Standard first-line	Lost to follow-up	Unsuccessful
ISO47	Susceptible	-	Sensible	Ser315Thr	Résistant	-	Sensible	INH-mono	Concordant (S)	Standard first-line	Treatment completed	Success

ISO48	Susceptible	-	Sensible	-	Sensible	-	Sensible	None	Concordant (S)	Standard first-line	Cured	Success
ISO49	Susceptible	-	Sensible	-	Sensible	-	Sensible	None	Concordant (S)	Standard first-line	Cured	Success
ISO50	Susceptible	-	Sensible	-	Sensible	-	Sensible	None	Concordant (S)	Standard first-line	Cured	Success
ISO51	Resistant	Asp435Val	Résistant	Ser315Thr	Résistant	-	Sensible	MDR (RIF+INH)	Concordant (R)	MDR-TB regimen	Cured	Success
ISO52	Susceptible	-	Sensible	-	Sensible	-	Sensible	None	Concordant (S)	Standard first-line	Cured	Success
ISO53	Susceptible	-	Sensible	-	Sensible	-	Sensible	None	Concordant (S)	Standard first-line	Cured	Success
ISO54	Susceptible	-	Sensible	-	Sensible	-	Sensible	None	Concordant (S)	Standard first-line	Cured	Success
ISO55	Susceptible	-	Sensible	-	Sensible	-	Sensible	None	Concordant (S)	Standard first-line	Treatment completed	Success
ISO56	Susceptible	-	Sensible	-	Sensible	Met306Val	Résistant	EMB-mono	Concordant (S)	Standard first-line	Cured	Success
ISO57	Susceptible	-	Sensible	-	Sensible	-	Sensible	None	Concordant (S)	Standard first-line	Cured	Success
ISO58	Susceptible	-	Sensible	-	Sensible	-	Sensible	None	Concordant (S)	Standard first-line	Cured	Success
ISO59	Susceptible	-	Sensible	-	Sensible	-	Sensible	None	Concordant (S)	Standard first-line	Cured	Success
ISO60	Susceptible	-	Sensible	-	Sensible	-	Sensible	None	Concordant (S)	Standard first-line	Cured	Success

ISO61	Susceptible	-	Sensible	-	Sensible	-	Sensible	None	Concordant (S)	Standard first-line	Cured	Success
ISO62	Resistant	Asp435Tyr	Résistant	Ser315Thr	Résistant	-	Sensible	MDR (RIF+INH)	Concordant (R)	MDR-TB regimen	Cured	Success
ISO63	Susceptible	-	Sensible	-	Sensible	-	Sensible	None	Concordant (S)	Standard first-line	Cured	Success
ISO64	Susceptible	-	Sensible	Ser315Thr	Résistant	-	Sensible	INH-mono	Concordant (S)	Standard first-line	Cured	Success
ISO65	Susceptible	-	Sensible	-	Sensible	-	Sensible	None	Concordant (S)	Standard first-line	Cured	Success
